# The Influence of Water Composition on Flavor and Nutrient Extraction in Green and Black Tea

**DOI:** 10.3390/nu11010080

**Published:** 2019-01-03

**Authors:** Melanie Franks, Peter Lawrence, Alireza Abbaspourrad, Robin Dando

**Affiliations:** Department of Food Science, Cornell University, Ithaca, NY 14850, USA; mf755@cornell.edu (M.F.); petegcms@gmail.com (P.L.); alireza@cornell.edu (A.A.)

**Keywords:** taste, sensory evaluation, tea, EGCG, hedonics

## Abstract

Tea is made from the processed leaves of the *Camellia sinensis* plant, which is a tropical and subtropical evergreen plant native to Asia. Behind water, tea is the most consumed beverage in the world. Factors that affect tea brewing include brewing temperature, vessel, and time, water-to-leaf ratio, and, in some reports, the composition of the water used. In this project, we tested if the water used to brew tea was sufficient to influence perceived flavor to the everyday tea drinker. Black and green tea were brewed with bottled, tap, and deionized water, with brewing temperature, vessel, time, and the water-to-leaf ratio matched. The samples were analyzed with a human consumer sensory panel, as well as instrumentally for color, turbidity, and Epigallocatechin Gallate (EGCG) content. Results showed that the type of water used to brew tea drastically affected sensory properties of green tea (and mildly also for black tea), which was likely driven by a much greater degree of extraction of bitter catechins in teas brewed with more purified bottled or deionized water. For the everyday tea drinker who drinks green tea for health, the capability to double the EGCG content in tea by simply brewing with bottled or deionized water represents a clear advantage. Conversely, those drinking tea for flavor may benefit from instead brewing tea with tap water.

## 1. Introduction

### 1.1. Tea and Tea Processing

Tea is a beverage steeped in culture and history. Valued for its taste and caffeine content as well as its numerous health properties [1], tea has been consumed for centuries [2]. Behind water, tea is the most consumed beverage in the world [3]. The botanical name for the plant producing tea is *Camellia sinensis* (L.) Kuntze. There are many other plants used for extraction such as rooibos and chamomile, however these are not strictly teas. Instead, they are classified under the category of tisanes or herbal infusions. The main difference between various styles of tea is the level of oxidation of the leaf during processing. Green and white teas are unoxidized, oolongs vary in the levels of oxidation, and black tea leaves are fully oxidized. A cup of tea is made from processed fresh tea leaves. Biochemical changes that occur during processing help reduce the bitter taste of fresh tea leaves. Processing the tea leaves lowers water content to aid in shelf stability, deactivates enzymes, and adds sweetness and a myriad of colors to the cup. Physically the leaf transforms from a sturdy crisp leaf to limp and pliable during withering. Chemically, caffeine content increases, hydrolysis of hydrophobic carbohydrates begins, non-gallated catechins and aroma compounds form, and the levels of chlorophyll and various enzymes increase [4]. For black teas, after withering, the leaves are purposefully crushed to speed oxidation. This step is what gives black tea its defining quality, whereby enzymatic oxidation converts catechins into theaflavins and thearubigins. Polyphenols give black tea its reddish-brown coloration [5]. 

### 1.2. Tea Flavanols

The main polyphenols found in tea are flavonoids. Flavonoids are a group of bioactive compounds synthesized during plant metabolism. Flavonoids are found in fruits and vegetables, prominently in spinach, apples, and blueberries, as well as in beverages like tea and wine. Previous health-related research on tea has largely focused on the flavonoid group. Flavonoids contain two six-carbon rings linked by a three-carbon unit, which is also known as a chalcone structure [6]. Catechins (also referred to as flavanols) are bioactive compounds that are a subclass of flavonoids, and, in tea, are the main secondary metabolites. The main catechins in tea are: catechin, epicatechin, epicatechin gallate, epigallocatechin, epigallocatechin-3-gallate, and gallocatechin. Catechin content in tea differs by tea type or style. Catechins in green tea are relatively stable since they do not go through oxidation during processing, and are what gives green tea its characteristic bitterness and astringency. In black tea, the catechins are largely oxidized to theaflavins and thearubigins [6], which reduces catechin content by around 85% compared to green tea [7], leaving the tea darker and less bitter. 

### 1.3. Tea and Water

After tea leaves are harvested and processed, the final product is ready to consume. However, unlike many other beverages, the final processing step is left to the consumer. A high-quality tea that has gone through many labor-intensive steps can be ruined in an instant by improper brewing. Factors that alter the taste of the brewed cup are brewing temperature, time, vessel, the water-to-leaf ratio, and the water composition [8,9]. This study focuses on the water used to brew tea, specifically how water quality influences the sensory and chemical qualities of black and green tea. Taste is a key factor in consumer acceptance of water [10], however water is often not a top priory when making tea, despite its critical role as the vehicle for the infusion. References to the importance of water content in brewing tea can be found as early as 758AD, in The Classic of Tea by Lu Yu [11]. Lu Yu was an orphan during the Tang Dynasty, raised by an abbot in the Dragon Cloud Monastery. He authored an efficient 7000-character book detailing how to harvest, process, and brew tea, including what types of water are suitable for tea, as well as the proper tools and utensils. Lu Yu felt that tea made from mountains streams was ideal, river water was sufficient, and well water was inferior [3]. In a more recent book from Kuroda & Hara [12], tap water is recommended as the most suitable water for making tea, although specific recommendations are that water should be clear of odors and deficient in magnesium and calcium.

Previous work suggests that tap water can influence the amount of tea flavanols extracted in green tea compared to brewing green tea with purified water [13]. Tap water has a differing (inconsistent between regions, and over time) mineral balance. “Hard” water is high in minerals such as calcium and magnesium. Tea infusions are particularly affected by calcium, with previous studies showing that levels of theaflavins and caffeine extracted decrease with high levels of calcium [14]. Magnesium and calcium can also promote two undesirable outcomes of tea brewing: tea cream and scum formation. Tea cream is the precipitate matter that forms as the tea cools and is caused by the reaction between caffeine and tea flavanols, while tea scum is a surface film that forms on the tea infusion surface, which is composed of calcium, hydrogen carbonates, and other organic material. This film occurs due to calcium carbonate triggering oxidation of organic compounds [9]. It has also been demonstrated that catechin extraction can be increased in white tea by brewing with purified water [15].

### 1.4. Tea Flavor

Between 25% to 35% of the fresh tea leaf is composed of phenolic compounds with 80% of these being flavanols [16]. Both phenolic compounds and alkaloids such as caffeine contribute to the bitter taste in tea, though the catechins are thought to be the main contributors to bitterness [17]. Glucose, fructose, sucrose, and arabinose in tea account for its sweet taste. Free amino acids make up about 1% to 3% of the dry leaf, and, in green tea, may yield an umami characteristic [16]. Astringency, albeit not a taste, is a common oral sensation in tea, thought to arise from its catechin content [18]. Despite tea being consumed for several thousand years, there are few consumer sensory studies of tea flavor, with researchers more often favoring evaluation by trained or expert panels. The goal of this project was to test if the water source used to brew tea (tap, bottled, or deionized) influenced flavor or liking from the everyday tea drinker, using both black and green tea. Tea samples were analyzed with a human consumer sensory panel as well as with a number of instrumental methods.

## 2. Materials and Methods

### 2.1. Mineral Analysis of Water Samples

Ithaca city tap water, Poland Spring^TM^ bottled water (Nestle Waters, Paris, France), and deionized water used for the study were tested by the Community Science Institute, Inc (Ithaca, NY, USA), assaying calcium, iron, magnesium, sodium, and copper content. Methods followed those recommended by the Environmental Protection Agency (EPA). Briefly, Iron, Magnesium, and Sodium were measured spectrochemically (EPA protocol 200.2, Rv. 2.8) and with inductively coupled plasma-atomic emission spectrometry (EPA 200.7, Rv 4.4), while copper was measured using Inductively Coupled Plasma Mass Spectrometry (EPA 200.8/EPA200.8, Rv 5.4). Calcium and residual chlorine were measured colorimetrically, using an EDTA titration for calcium (SM 3500-Ca B), and a Lamotte test kit for chlorine (LaMotte DPD-1R, LaMotte Co., Maryland, USA).

### 2.2. Preparation of Tea Infusions

Two high-quality loose leaf teas known as Zhejiang green and Mao Feng black teas were purchased from In Pursuit of Tea (New York, NY, USA). Both teas are from the Zhejiang Province in China, which is a highly regarded tea region, with both produced on the same farm. Green teas were brewed in tap (GT) water, bottled (GB) water, and deionized (GD) water, with black similarly denoted as black tea in tap (BT), bottled (BB), and deionized (BD) water. For the green tea samples, 2.5 g of tea was weighed out into pre-warmed Gaiwan tea brewing vessels (Figure S1), with 125 mL of water at 80 °C added to the vessel. The green tea infusion was brewed for three minutes and then strained through a fine mesh strainer. Black tea samples were brewed at 100 °C for 5 min (more typical for black tea preparation), and strained. Samples were then either cooled to room temperature for instrumental analysis or served fresh in pre-heated cups for sensory analysis (see 2.6 below).

### 2.3. Colorimetry

Analysis of tea color was performed with a Hunter Lab UltraScan VIS colorimeter (Reston, VA, USA). L (light vs dark), a (red vs green), and b (yellow vs blue) values were recorded for each sample with each of the samples measured in triplicate. 

### 2.4. Turbidity

The turbidity of each sample was measured in triplicate with use of a HACH 2100P portable Turbidity meter (Loveland, CO, USA), with measurements recorded in Nephelometric Turbidity Units (NTU). The samples were held at a 90° angle to the incident beam using single detection. Turbidity standards used were 0.1 NTU, 20 NTU, and 100 NTU. 

### 2.5. Analysis of EGCG

Epigallocatechin Gallate (EGCG) in the tea infusions was measured using high performance liquid chromatography (HPLC), following the methods of Wang and Helliwell [13]. Samples were run using an Agilent 1100 HPLC system (Santa Clara, CA, USA) with a DAD detector. Separations were carried out using a Waters Cortecs (Milford, MA, USA) C18 (4.6 mm × 100 mm) column using an isocratic solvent system consisting of 90% 0.01% phosphoric acid in Millipore water (*v*/*v*) and 10% methanol with a flow rate of 0.6 mL/min. The column was held at a constant temperature of 30 °C. The DAD detector was set to 210 nm. Sample injection volume was 10 µL. The total run time was 20 min. All samples were filtered just before being loaded onto the HPLC using a 0.22 µm Polyvinylidene Fluoride (PVDF) filter from Celltreat (Pepperell, MA, USA). Quantification was performed by the use of an external standard curve using purified EGCG purchased from Sigma Aldrich (St Louis, MO, USA). Identification of EGCG in tea samples was performed using retention time of the pure standards (10.26 min).

### 2.6. Sensory Evaluation

**Figure 1:**
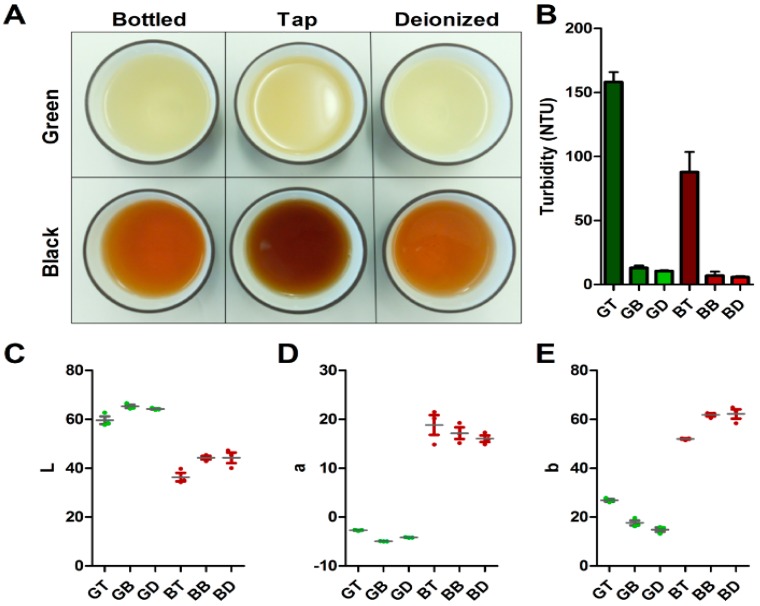
(**A**) Image of black and green tea samples brewed in tap, bottled, or deionized water. For both green and black tea, infusions appear darker and cloudier from tap wate compared to the teas brewed in DI or bottled water. (**B**)Turbidity measurements (NTU) for each tea infusion showing average of three replicates with SEM. (**C–E**) Colorimeter readings from tea infusions, L, a and b values displayed with individual readings as dots, lines denoting average, and SEM. Samples denoted as green tea brewed in tap (GT), bottled (GB), and deionized (GD) water, black tea brewed in tap (BT), bottled (BB), and deionized (BD) water. Green tea samples represented in green, black tea in dark red.

All human study procedures were approved by the Cornell University Institutional Review Board for Human Participants, with all methods performed in accordance with relevant guidelines and regulations. A total of 103 panelists were recruited from the local community, pre-screened for their tea drinking behavior, and all gave informed consent. All the participants in the study drank tea three to five times a week or more, and were both green and black tea drinkers. The panelist either habitually consumed tea with no milk or sugar added to it or stated no dislike of tea in this manner. Participants knew that the study involved tea but were unaware of the true objective of the research. The session took approximately 45 min, with panelists compensated for their time. The panelists answered questions about samples in individual booths, using Red Jade sensory evaluation software (Curion, Deerfield, IL, USA). The samples were delivered monadically, in a counterbalanced full-block design, but panelists either received 3 green tea samples or 3 black tea samples first. Each tea sample was evaluated for overall liking, appearance liking, and flavor liking with 9-point scales, and then used the generalized Labeled Magnitude Scale (gLMS) to test sweetness, bitterness, sourness, astringency, vegetal quality (for green tea only), and earthiness (for black tea only). All panelists were briefly trained on how to use the gLMS before beginning the tasting [19]. The color of the tea was also evaluated by panelists with a color matching sheet (Figure S2) from which they chose the closest match for each tea sample. Teas were freshly brewed every 30 min. A total of 10 g of tea was brewed with 500 mL of water, at 80 °C for green tea, and 100 °C for black tea. All infusions were kept warm in pre-heated, insulated carafes until the panelist was ready for the sample. Samples were served in pre-heated (80 °C) white ceramic Gung Fu cha teacups (see Figure 1 below) labeled with random 3-digit codes. After each sample, panelists were instructed to cleanse their palette with water and non-salted crackers to avoid fatigue as well as deter any lingering bitterness or astringency. At the end of the questionnaire, panelists were asked a series of demographic questions and for information on their tea drinking habits. 

### 2.7. Statistical Analysis

Data were analyzed with repeated measure analyses of variance (ANOVA) and post-hoc Tukey’s tests using Graphpad Prism 5.0 (Graphpad Software, La Jolla, CA, USA). Separate ANOVAs were used for green and black tea samples since such large differences in taste and chemical properties have been shown previously. Statistical significance was inferred at *p* < 0.05. Multivariate analysis was performed using XLSTAT (Addinsoft, Paris, France) whereby two separate Principal Components Analyses were run on sensory and instrumental data as well as these two datasets combined in a Multiple Factor Analysis.

## 3. Results and Discussion

### 3.1. Water Analysis

Deionized, tap, and bottled water samples were tested for calcium, magnesium, copper, iron, residual chlorine, and sodium (Table 1). The amount of calcium, magnesium, and sodium in tap water was far greater than that in bottled or deionized water. 

### 3.2. Turbidity and Color

Figure 1A shows the appearance of tea samples when brewed with three different water types. Teas brewed in tap water appear more cloudy and darker in color than teas brewed in bottled water or deionized (DI) water for both green and black teas. Turbidity measurements (Figure 1B) in green (*p* < 0.001) showed GT was more turbid than both GB (95% CI = 133.3 to 156.7) and GD (95% CI = 135.7 to 159.1), with no difference between GB and GD. In black tea, the turbidity of BT was also higher (*p* < 0.001) than both BB (95% CI = 57.66 to 103.9) and BD (95% CI = 58.81 to 105.1), with no difference between BB and BD. Adding high concentrations of calcium or magnesium in water can cause cloudiness and tea scum in tea infusion as well as possibly influencing tea’s sensory properties [9,18] since both calcium and magnesium were higher in tap water used in this project. This was likely the cause of the observed turbidity increase.

Both green (*p* = 0.016) and black (*p* = 0.023) tea infusions significantly differ in lightness. Green tea brewed in tap water exhibited lower L values compared to the same tea brewed in bottled (95% CI = −9.992 to −1.288) or DI (95% CI = −8.952 to −0.2476) water, with BT similarly lower than BB (95% CI = −15.14 to −0.7051) or BD (95% CI = −15.13 to −0.6918). The a values for green (*p* < 0.001) but not black (*p* = 0.425) tea significantly differed between samples, with all pairs differing between green teas (95% CI for GT vs GB = 2.042 to 2.458; GT vs GD = 1.269 to 1.685; GB vs GD = −0.9814 to −0.5652). The b values for both green (*p* < 0.001) and black (*p* = 0.001) teas significantly varied between treatments, with tap water against the different sample. GT was higher compared to GB (95% CI = 5.661 to 12.80) and GD (95% CI = 8.401 to 15.540), with BT higher than BB (95% CI = −14.94 to −4.711) or BD (95% CI = −15.33 to −5.105).

### 3.3. EGCG Content

**Figure 2:**
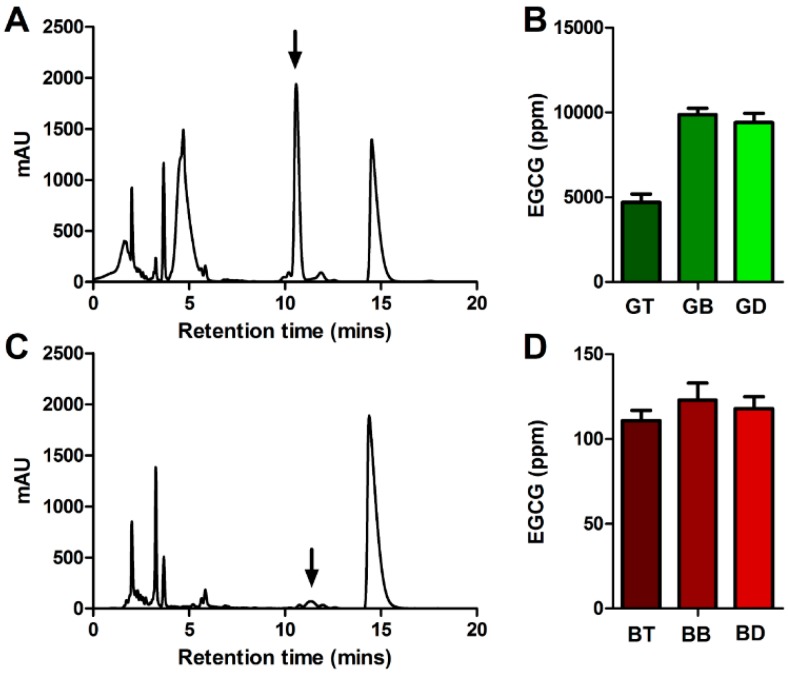
(**A**) Chromatogram illustrative of HPLC spectrum from green tea. EGCG peak at arrow. Y axis in milli-Absorbance Units. (**B**) Total EGCG content for green tea in ppm, brewed in tap (GT), bottled (GB), and deionized (GD water. Display shows mean of three readings plus SEM. (**C**) Chromatogram illustrative of HPLC spectrum from black tea. EGCG peak at arrow. (**D**) Total EGCG content for black tea in ppm. Samples denoted as black tea brewed in tap (BT), bottled (BB), and deionized (BD) water.

The amount of EGCG in black tea is customarily lower than that found in green tea, since the majority of the catechins in black tea are converted to theaflavins and thearubigins [5]. The small amount of EGCG in the black tea infusions did not vary with water type (*p* = 0.250, Figure 2C,D). Conversely, with green tea (natively much higher in EGCG), there was a significant difference between green tea infusions (*p* < 0.001) and with green tea brewed in bottled water (95% CI = −6350 to −3984) and in deionized water (95% CI = −5890 to −3524) having around double the amount of EGCG compared to green tea brewed in tap water (Figure 2A,B), despite being brewed from the same leaves, at the same strength, time and temperature, in identical vessels. Green teas brewed from bottled or deionized water achieved around the same level of EGCG extraction (95% CI = −723.0 to 1643). Such dramatically inferior EGCG extraction in tap water is important to green tea consumers, many of whom are consuming green tea due to a perceived consequence of health promotion [20]. EGCG is the most abundant catechin in green tea [21] as well as one of the most bitter tasting [22]. That green tea acceptance has been linked to bitter taste genes [23], and that bitterness in tea is largely a product of EGCG content [24], implies that extraction of bitter catechins in bottled or deionized water may lead to more healthy and yet less palatable tea infusions.

### 3.4. Sensory Testing of Tea Samples

**Figure 3:**
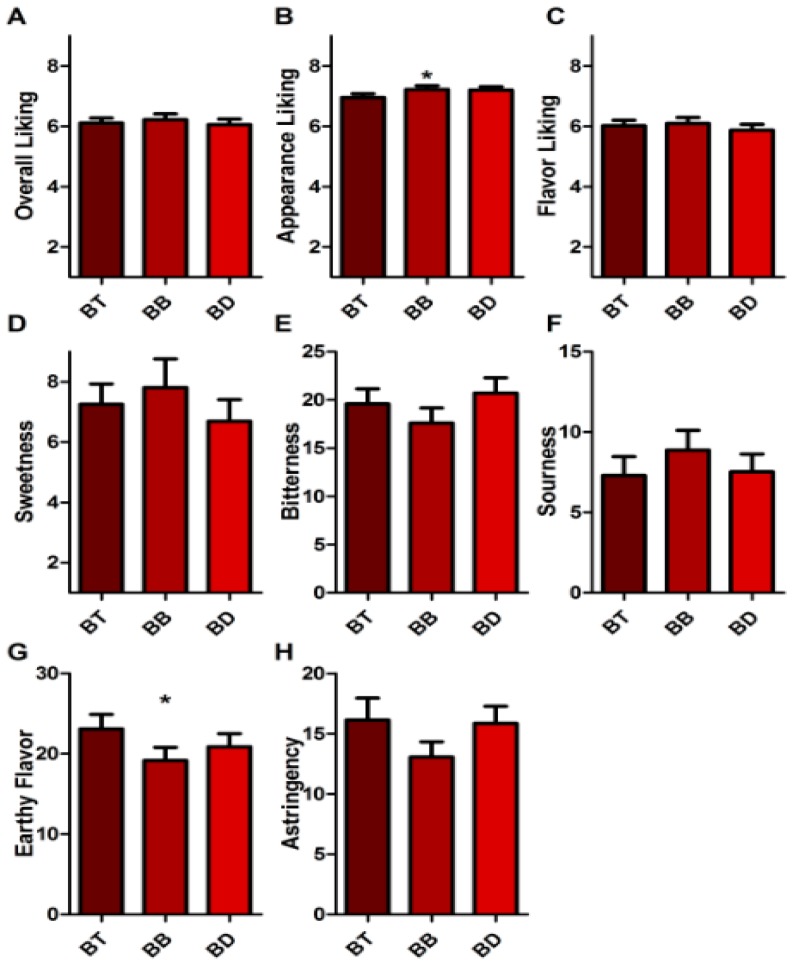
Consumer perception of black tea brewed in tap (BT), bottled (BB), and deionized (BD) water. (**A**) Overall liking of samples, from dislike extremely (1) to like extremely (9). (**B**) Appearance liking of samples, from dislike extremely (1) to like extremely (9). (**C**) Flavor liking of samples, from dislike extremely (1) to like extremely (9). (**D**) Perceived sweetness of samples, rated on gLMS, scale descriptors no sensation (0.0), barely detectable (1.4), weak (6.0), moderate (17.0), strong (34.7), very strong (52.5), and strongest imaginable sensation of any kind (100.0). (**E**) Bitterness, scale as in D. (**F**) Sourness, scale as in D. (**G**) Earthy flavor, scale as in D. (**H**) Astringency, scale as in D. Bars display mean rating of panel (*n* = 103) plus SEM. * indicates *p* < 0.05.

There was no significant difference between panelists’ overall (*p* = 0.646), or flavor (*p* = 0.553) liking of black tea samples (Figure 3A,C). Panelists did find significant differences in appearance liking between the samples (Figure 3B, *p* = 0.0345), which is likely a reflection of the color differences between the black tea infusions evident in Figure 1A. However, this trend was not strong enough to reflect differences between sample pairs in post-hoc Tukey’s tests. Panelists also evaluated various flavor attributes of the black tea infusions. No differences were evident with water type between black tea infusion for astringency, bitterness, sourness, or sweetness (Figure 3D–F,H, all *p* > 0.05). However, panelists did find a difference in earthy flavor (Figure 3G, *p* = 0.025), specifically between that brewed in bottled water compared to black tap water (95% CI = −7.339 to −0.5252). While the panel perceived black tea brewed in tap water to be earthier, it had little effect on liking, which suggests that water may not be a critical factor in determining liking in black tea.

**Figure 4:**
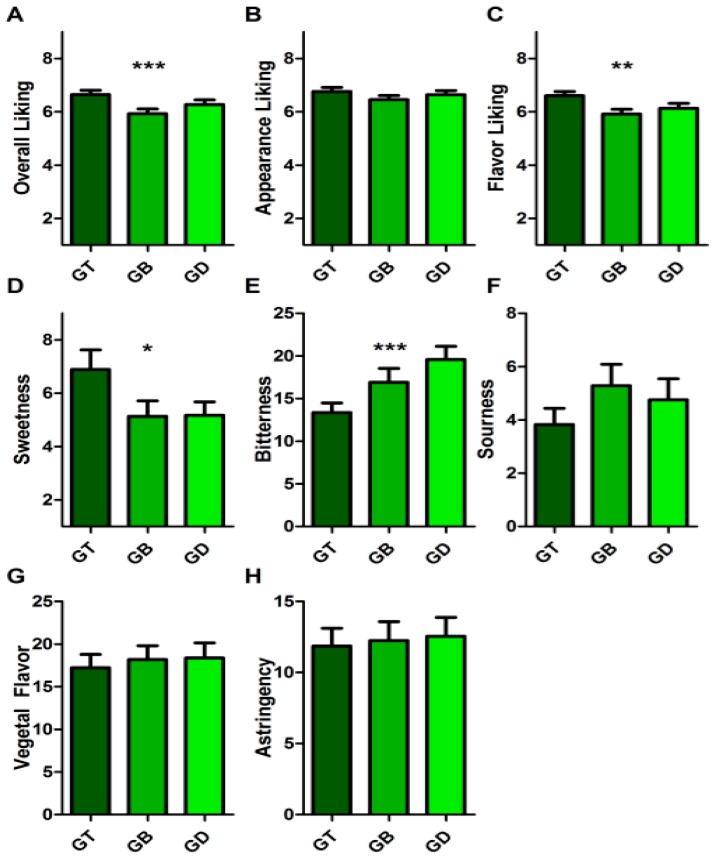
Consumer perception of green tea brewed in tap (GT), bottled (GB), and deionized (GD) water. (**A**) Overall liking of samples, from dislike extremely (1) to like extremely (9). (**B**) Appearance liking of samples, from dislike extremely (1) to like extremely (9). (**C**) Flavor liking of samples, from dislike extremely (1) to like extremely (9). (**D**) Perceived sweetness of samples, rated on gLMS, scale descriptors no sensation (0.0), barely detectable (1.4), weak (6.0), moderate (17.0), strong (34.7), very strong (52.5), and strongest imaginable sensation of any kind (100.0). (**E**) Bitterness, scale as in D. (**F**) Sourness, scale as in D. (**G**) Earthy flavor, scale as in D. (**H**) Astringency, scale as in D. Bars display mean rating of panel (*n* = 103) plus SEM. * indicates *p* < 0.05. ** indicates *p* < 0.01. *** indicates *p* < 0.001.

For green tea samples, the effects of water were clearer. Panelists rated their overall liking (Figure 4A, *p* < 0.001) of green tea samples as differing across water treatments, with the tap clearly higher than bottled water (95% CI = −1.138 to −0.2993), with tap vs. deionized water approaching significance (95% CI = −0.04054 to 0.7978). Interestingly, this reduction in liking seemed to be driven by the panel’s liking of the sample’s flavor (Figure 4C, *p* = 0.001), and not its appearance (Figure 4B, *p* = 0.099). In investigating changes to the green tea’s flavor properties, panelist found no significant difference in astringency, sourness, or vegetal flavor (Figure 4F–H, all *p* > 0.05). However, the panel judged the green tea samples brewed with tap water to be far less bitter (Figure 4E, *p* < 0.001) than both the sample brewed with bottled (95% CI = 0.6244 to 6.502) or with deionized water (95% CI = −9.162 to −3.285). Since only around half the amount of EGCG was extracted in green tea brewed from tap water compared to the other samples, and EGCG is experienced as highly bitter, this would result in less bitter tea infusion when brewing with tap water. Since bitterness is closely linked to liking tea regardless of ethnicity or tea drinking habits [25,26], this likely drove the increase in liking of green tea brewed in tap water. The GT sample was also experienced as sweeter by the panel (Figure 4D, *p* = 0.012), which was likely due to mixture suppression [27,28] of sweetness in samples with more bitter catechins. EGCG has been noted to extract more efficiently from green tea with purer water [29] and with higher conductivity (thus higher impurity) water producing poorer catechin extraction [30]. Rossetti and colleagues [31] measured the detection threshold of EGCG (perceived to be bitter and astringent) to be 183 mg/L (at 37 °C). Despite the fact that bitterness may be somewhat depressed by temperature [32], the bitterness of green tea in our study would be clear in the samples’ flavor profile. Thus, doubling the EGCG content of tea in bottled or deionized water (compared to tap) was likely the driving factor behind reduced liking of these samples in consumer testing. Since black tea has fewer catechins than green tea due to the oxidation process in manufacturing, the type of water used seems less important to the everyday tea drinker.

As well as instrumental measurement of color changes in tea samples, and assessment of appearance liking of samples, we were also interested in whether variation in color between samples was visible to the human eye. Panelists used a color matching chart for both black and green tea samples (see Figure S2), divided into eight color segments for green teas, and eight more for black teas. The panelists could clearly discern differences between samples of both black (*p* < 0.001, chi-square 39.91), and green tea samples (*p* < 0.001, chi-square 43.87), although this did not influence their liking of the samples overall, nor their liking of the appearance of the samples, which suggests flavor is more critical in determining liking of tea infusions than their appearance. It is clear, however, that consumer perception of beverages can be altered by their color and appearance [33], and, thus, some of the effects observed may have been due to the cross modal influence of the different colored tea samples.

Some work exists concerning the influence of various brewing conditions on the sensory properties of tea. Liu et al. [34] found optimal conditions for acceptance, at least in a small expert panel, were brewing for 5.7 min at 82 °C, with tea of around 1100 µm in particle size, in a 70 mL/g ratio of water to tea. From instant green tea preparations, increasing the calcium concentration in the brewing solution was found to weaken bitter taste in the mixture purportedly provided by EGCG [18], which is in good agreement with our observations. However, influences on the sweetness of infusions (attributed in part to theanine) were not seen in our work, possibly due to the around 4 mg/100 mg sucrose found in the group’s instant tea preparations. A study of hot and cold-brewed tea infusions of varying strength by Lin et al. [2] proposed a linkage between higher EGCG and EGC (epigallocatechin) levels and lower sensory appeal, which was attributed to lower bitterness and astringency in these samples. In a small group of trained panelists, sensory differences were reported in green tea brewed with various water types [30], with mineral water found to produce tea with lower EGCG levels than tap water, purified water, or mountain spring water, as well as perceived bitterness mapping onto EGCG levels. However, samples from this report were liked more with higher bitterness (and EGCG) unlike our own results. A similar result was reported by Zhang et al. [15], whereby EGCG levels from green tea extractions varied with water quality. Sensory reports of taste quality were higher for the high EGCG samples, though no report was made of panel size or makeup. Such differences are likely attributed to the difference in palate of a small group of experts from China versus a large panel of tea consumers in the US. Alternatively, those regularly consuming diets high in salty [35], sweet [36], or umami [37] stimuli have shown some reduced ability to perceive these stimuli possibly due to receptor regulation in taste [38]. Thus, it is possible that regular consumers of very bitter tea experience how they taste in a fundamentally different manner.

Following sensory testing, panelists participated in a survey of their attitudes toward tea. When asked their primary motivation for drinking black tea, only 7% of panelists responded due to healthful properties and, instead, favoring taste or flavor (84%), with a small number of respondents citing other reasons. However, when asked their primary motivation for drinking green tea, 26% cited its health benefits, with 67% for taste or flavor, and again a small number citing other reasons. This suggests the ability to almost double the EGCG content of green tea would be of great interest to many green tea consumers.

### 3.5. Multivariate Analysis

**Figure 5:**
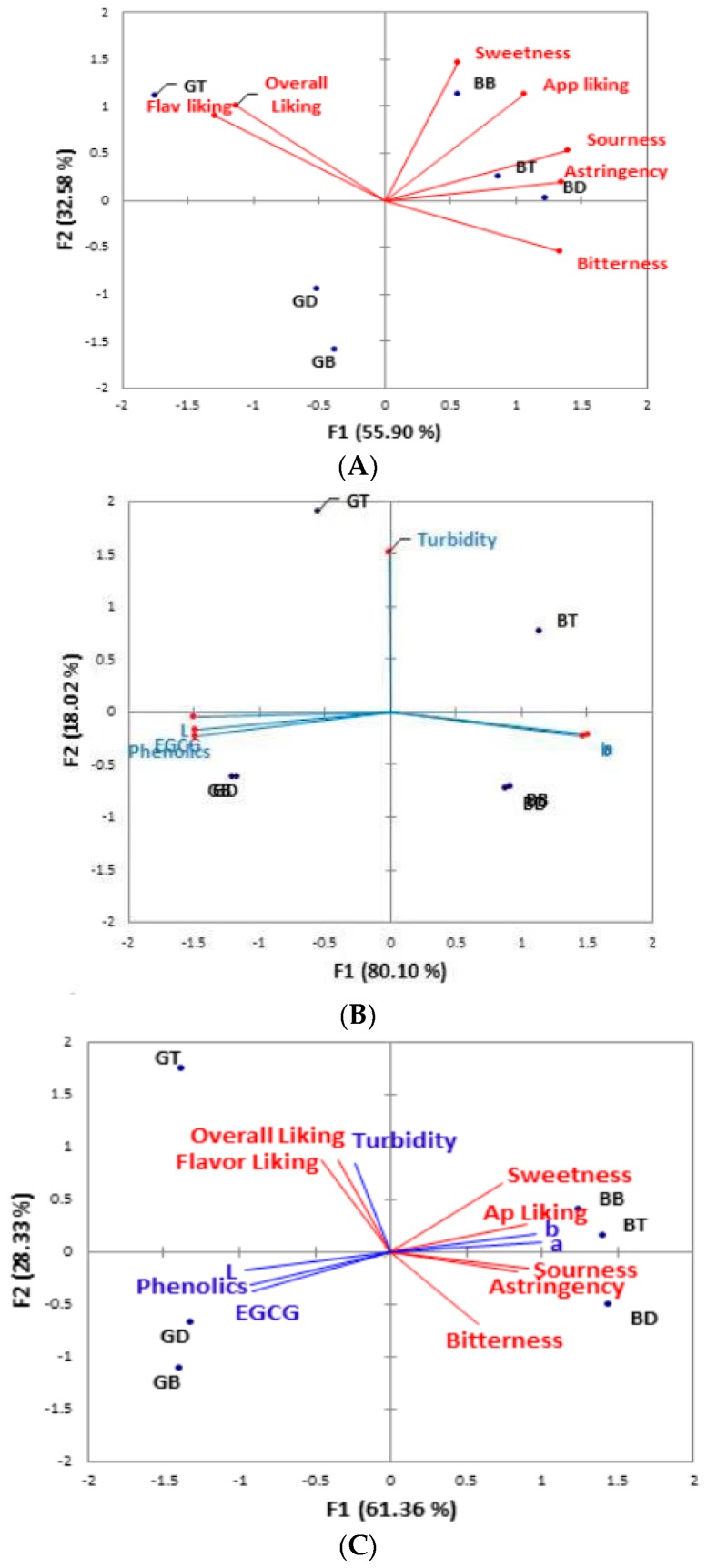
Multivariate analysis of tea samples. (**A**) Principal components analysis of sensory data. Samples shown in black, original axes in red, variance from new factors in parentheses. (**B**) Principal components analysis of instrumental data. Samples shown in black, original axes in blue, variance from new factors in parentheses. (**C**) Multiple factor analysis of sensory and instrumental data. Samples shown in black, sensory axes in red, instrumental axes in blue, variance from new factors in parentheses. Samples denoted as green tea brewed in tap (GT), bottled (GB), and deionized (GD) water, black tea brewed in tap (BT), bottled (BB), and deionized (BD) water.

Further analysis of the data with Principal Components Analysis (PCA) and Multiple Factor Analysis (MFA) was performed. Scree plots revealed that data could be plotted well on two axes both in the case of sensory and instrumental data, with 88.5% and 98% of the variance accounted for by the first two factors in the analysis, respectively. The sample of green tea brewed with tap water was located close to both dimensions of overall and flavor liking, which, in turn, were negatively correlated with bitterness (Figure 5A). In plots of instrumental results, samples pairs GD and GB as well as BD and BB plotted almost exactly on top of one another (Figure 5B). In the case of both black and green tea, the tap-brewed sample was the clear outlier. Samples GD and GB plotted closely to the axes represent phenolics, EGCG, and colorimetric L-value. MFA plots combining both sensory and instrumental data showed similar patterns (Figure 5C), with sample GT lying in the directions of overall and flavor liking, and anti-parallel to that of bitterness. 

## 4. Conclusions

Tea is the most consumed beverage besides water in the world. This project sought to get a better understanding of whether the type of water used to brew tea is of importance to the everyday tea drinker. Through the instrumental analysis of green and black tea brewed in tap, bottled, and deionized water, we demonstrated a difference in color, turbidity, and the amount of EGCG extracted from tea leaves depending on the water type. The high mineral content of the tap water used in this study led to inferior extraction of catechins in green tea, and thus, produced an infusion that was less bitter, and also perceived as sweeter than the same tea brewed in bottled or deionized water, with an accompanying higher degree of liking for green tea when brewed in this manner. For tea drinkers consuming green tea for either flavor or its health benefits, our results highlight that the type of water used to brew tea is clearly important, and suggests that those seeking greater health benefits should use a more purified water source to brew green tea, while those more concerned with flavor may prefer to use water from the tap.

## Figures and Tables

**Table 1:** Mineral analysis of the different water types in mg/L.

	Bottled	Tap	Deionized
Calcium	8.000	53.600	3.000
Iron	0.050	0.050	0.050
Magnesium	1.370	9.460	0.100
Sodium	10.600	20.900	0.100
Copper	0.002	0.176	0.002
Residual Chlorine	0.200	0.200	0.200

## Supplementary Materials

The following are available online at http://www.mdpi.com/2072-6643/11/1/80/s1, Figure S1: Traditional Gaiwan brewing vessel, Figure S2: 8-option color matching diagram provided to consumer panel.
