# Supplementary figures and images for: The Influence of Water Composition on Flavor and Nutrient Extraction in Green and Black Tea
Source: Nutrients. 2019 Jan 3;11(1):80. doi: 10.3390/nu11010080 (PMC6356489; doi:10.3390/nu11010080)

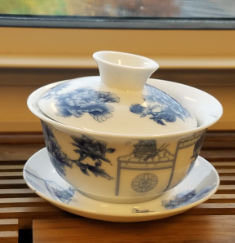

Supplement: Supplementary file 1 [file nutrients-11-00080-s001.zip › SF1.tif]

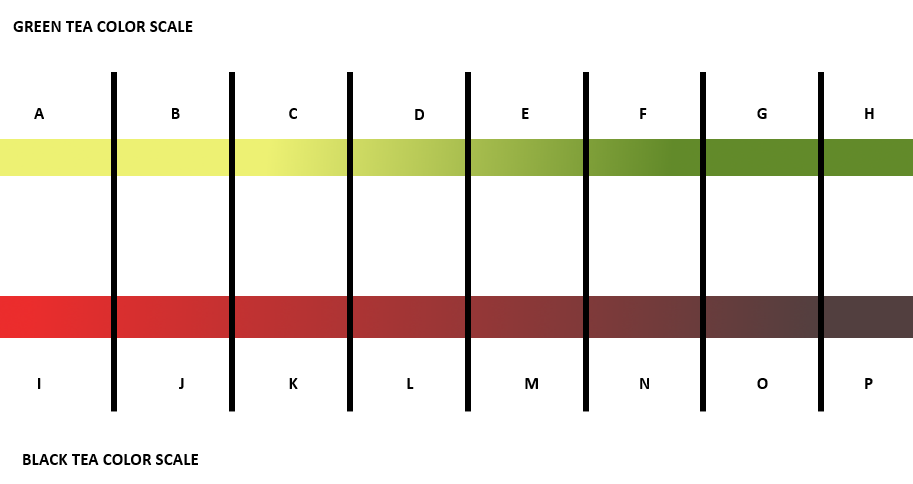

Supplement: Supplementary file 1 [file nutrients-11-00080-s001.zip › SF2.tif]
